# The importance of long‐term experiments in agriculture: their management to ensure continued crop production and soil fertility; the Rothamsted experience

**DOI:** 10.1111/ejss.12521

**Published:** 2018-01-18

**Authors:** A. E. Johnston, P. R. Poulton

**Affiliations:** ^1^ Sustainable Agriculture Sciences Department Rothamsted Research West Common, Harpenden AL5 2JQ UK

## Abstract

Long‐term field experiments that test a range of treatments and are intended to assess the sustainability of crop production, and thus food security, must be managed actively to identify any treatment that is failing to maintain or increase yields. Once identified, carefully considered changes can be made to the treatment or management, and if they are successful yields will change. If suitable changes cannot be made to an experiment to ensure its continued relevance to sustainable crop production, then it should be stopped. Long‐term experiments have many other uses. They provide a field resource and samples for research on plant and soil processes and properties, especially those properties where change occurs slowly and affects soil fertility. Archived samples of all inputs and outputs are an invaluable source of material for future research, and data from current and archived samples can be used to develop models to describe soil and plant processes. Such changes and uses in the Rothamsted experiments are described, and demonstrate that with the appropriate crop, soil and management, acceptable yields can be maintained for many years, with either organic manure or inorganic fertilizers.

**Highlights:**

- Long‐term experiments demonstrate sustainability and increases in crop yield when managed to optimize soil fertility.
- Shifting individual response curves into coincidence increases understanding of the factors involved.
- Changes in inorganic and organic pollutants in archived crop and soil samples are related to inputs over time.
- Models describing soil processes are developed from current and archived soil data.

## Introduction

Current issues about soil degradation and food production are not new, they are mirrored in Sanskrit literature (3500–4000 bc): “Upon this handful of soil our survival depends. Husband it and it will grow our food, our fuel and our shelter and surround us with beauty. Abuse it and the soil will collapse and die taking man with it”. Indeed, when they could no longer produce food many ancient civilizations died, like the Sumerian society in Mesopotamia, when its irrigated soils became too saline, and the Mayan society in Mesoamerica from feuding about responsibility for cultivating the terraced fields. Later, the Irish potato famine led to a mass exodus of people seeking new lands and opportunities to feed themselves. And very recently in the UK it has been suggested that soil fertility is declining to such an extent that there may be less than 100 harvests remaining! Little wonder then that today in many parts of the world there are widespread demands for the adoption of agricultural systems that are ‘sustainable’ and can continue to produce sufficient food, feed, fibre and biomass to support an existing and expanding human and animal population. However, it is not easy to define a ‘sustainable’ agricultural policy. At its most fundamental level it implies a system in which the quantity and quality of agricultural yield can be maintained year after year, that is financially viable for farmers and the local industries dependent on them, and does not cause degradation of the soil, environmental contamination, disruption of habitats for flora and fauna, pollution of water courses, and so on. Thus, sustainable agriculture should “meet the needs of the present without compromising the ability of future generations to meet their needs” (The Brundtland Report, Brundtland, 1987). Some issues like soil erosion and the transfer of phosphorus (P) from agricultural soil to surface water bodies are addressed best at the landscape scale, but it is our contention that answers to others are best obtained from long‐term field experiments with appropriate treatments. Such experiments, although not perfect, are the only practical way of assessing the long‐term sustainability and productivity of husbandry systems within the agro‐ecological zone in which they exist.

There are many long‐term field experiments worldwide, but probably too few to measure continuing food production in all the different types of soil and climate. The majority are in Europe and North America; for example, at Rothamsted and Woburn (UK), Askov (Denmark), Grignon (France), Bad Lauchstädt (Germany), and the Morrow plots and Sanborn Field (USA), and there have been others for different lengths of time in Asia, China and Australia, with fewer in South America and Africa. Many are listed in various databases (see e.g. http://iscn.fluxdata.org/partner‐networks/long‐term‐soil‐experiments) and Steiner (1995). Most of the older experiments were started to provide information on the amounts of nutrients and forms of fertilizer to use to increase crop yields. With time, new avenues of research have been developed within existing experiments to justify their continuation without compromising the aims of the original experiments. Examples include a better understanding of plant nutrition and soil fertility, and the damaging effects of weeds, and soil‐borne pests and diseases, and how they might be controlled as a wider range of agrochemicals has been introduced. But now, as costs escalate, funding them becomes ever more problematic, and long‐term experiments must now be modified or designed to examine those factors affecting food security, whilst preserving the integrity of the soil, and resolve environmental issues that threaten it.

The value of well‐designed and well‐executed experiments increases with time, as does their cost. However, cost effectiveness is increased if they serve more than one objective and are on well‐characterized sites that enable results to be extrapolated as widely as possible. Among such objectives are:

1. To test the sustainability of a farming system in an agro‐ecological zone over a long‐time span; to monitor the effects of climate change, including increasing atmospheric temperature, and make changes, if needed, to maintain sustainability.
2. To provide data to improve best husbandry practices to benefit farmers, local ecology and the wider environment.
3. To provide ongoing experiments with differently managed systems and an archive of soil and plant material to further scientific research into processes that control soil fertility, plant productivity, crop quality, and water and habitat quality.
4. To allow a realistic assessment of the effect of agricultural processes on the environment and of non‐agricultural anthropogenic activities on soil fertility and plant quality.
5. To provide long‐term datasets that can be used to develop mathematical models to describe a range of agricultural practices that could be used, for example, to predict the effects of climate change on soil properties and the productive capacity of soils.

Here we illustrate some of these objectives with examples from long‐term experiments managed by Rothamsted Research on silty clay loam at Rothamsted and sandy loam at Woburn (Chromic Luvisol and Cambic Arenosol, respectively; IUSS Working Group WRB, 2015). Average annual rainfall is 700 mm at Rothamsted (longitude 0°21′W; latitude 51°49′N) and 650 mm at Woburn (longitude 0°36′W; latitude 52°2′N), and at both sites the mean annual temperature of *c*. 9°C has increased by *c*. 1°C since the late 1980s (Scott *et al*., 2015). To achieve many of these objectives it has been necessary to manage the experiments actively, introduce appropriate modifications after careful consideration of the possible consequences, and importantly not allow the experiments to become unchanging monuments to their founders. We also refer to some experiments that have not continued and suggest reasons why this has happened. Some additional results and photographs of the long‐term experiments and the Sample Archive are in the Supporting Information.

## The start of the Rothamsted experiments

The scientific insight that has been gained into crop production, plant nutrition and soil fertility at Rothamsted owes much to the foresight shown by the station's founder, John Bennet Lawes, and his scientific collaborator for 57 years, Joseph Henry Gilbert. Not only did they start a number of long‐term field experiments between 1843 and 1856 but they retained them after they had achieved their initial aim. Importantly, Lawes & Gilbert also maintained a database of the results and an archive of crop and soil samples from each experiment.

In 1843, Lawes started his first two large field experiments on winter wheat (*Triticum aestivum* L.) on Broadbalk and turnips (*Beta vulgaris* L.) on Barnfield with treatments based on his earlier small‐scale experiments and those of Professor Daubeny (see Supporting Information). He also appointed Gilbert to assist him with their management. They realized quickly the need to develop their abilities to plan, design and supervise large‐scale field experiments and the importance of training their farm staff in appropriate experimental skills. Other large‐scale field experiments were started between 1847 and 1856, but, in most, only one crop was grown to understand its nutrient requirements better; the exception was the Agdell experiment where arable crops were grown in rotation (Johnston, 1994; Rothamsted Research, 2006). Some of these experiments continued for many decades and several still continue (Rothamsted Research, 2006).

Evidence in the Rothamsted archives suggests that Lawes contemplated stopping the experiments because of their cost, once the responses by the different crops to N, P and K had been established, and that appropriate amounts of fertilizers could give the same yield as 35 t ha^−1^ farmyard manure (FYM). That they continued is probably because of the controversy between Lawes and Gilbert in England and Liebig in Germany, which lasted for some 20 years, on the source from which plants derive their N (Johnston, 1990) (see Supporting Information). Much later, Lawes (Lawes, 1882) noted that field experiments should continue for long periods to get reliable conclusions because of the large annual variations in yield due to weather. He also commented that to explain some observations would require the aid of several branches of science (a multidisciplinary approach). Interestingly, he noted that to maintain financial support for experiments there is a danger that reports about them must contain “something of a sensational character” (Lawes, 1882), perhaps still a major danger!

### The archive of yield data, crop and soil samples and analytical techniques

Lawes and Gilbert's initial interest was in the response of crops to N, so that each year a sample of each crop grown on each plot was taken and analysed, and less frequently, soil samples were taken for analysis. These soil samples were taken by driving a metal box, 6 inches × 6 inches × 9 inches deep (15.2 cm × 15.2 cm × 22.9 cm), into the soil and then taking the soil from inside the box; this usually gave between 5 and 10 kg of soil and much of this still exists. We do not know why the soil was sampled to 23 cm (9 inches) when ploughing with oxen was to about 10 cm only, but as plough depth has increased to *c*. 20–23 cm sample depth has remained at 23 cm so that analytical soil data through the period of the long‐term experiments is comparable. Currently, soils are sampled more regularly with a semi‐cylindrical auger, bulking 15–25 cores per plot: about 400 g of soil is archived. The crop and soil samples now form a part of the unique archive going back to the mid‐19th century. In 1896, when Lawes & Gilbert were asked to put in order of importance the eight existing field experiments, they said that all were of equal importance. But importantly, Lawes also noted: “But, looking forward to the great questions which are being raised in regard to exhaustion of soil and restoration of fertility … that their soil samples and their history took first place in importance” (Anon., 1896). This has proved to be the case, especially where they have been used to follow trends over time in soil constituents.

Over the years, analytical techniques change or new ones are introduced. When analysing samples from long‐term experiments, great care is taken to ensure that data from a modified technique are directly comparable to the previous version, usually by analysing a wide range of samples with both techniques. If the two techniques are not directly comparable it may be possible to calculate an appropriate factor to convert from one to the other. Alternatively, if archived samples exist, these can be analysed retrospectively with new techniques. This assumes that there is no evidence to suggest that prolonged storage affects the property being measured. Ideally, samples should be stored under well‐controlled conditions.

In addition to the crop and soil samples, yield and soil data, together with analytical data, are kept at Rothamsted together with a print copy of all published papers. The handwritten and print archive is formidable but, unfortunately, one that is not easily accessible. To improve access, much of this information, including meteorological data, is now available in the electronic Rothamsted archive (e‐RA) maintained by the archivist curator (www.era.rothamsted.ac.uk), to whom enquires about access to the archive should be addressed.

### Two important results from the early years of the Rothamsted experiments

The two most important early results from the Rothamsted experiments were: (i) that crops did not respond to N when there was too little plant‐available P in the soil, and that more than one amount of N should be tested, and (ii) that inorganic fertilizers (with the ‘right’ amount of N) gave the same yield as 35 t ha^−1^ FYM, then the major source of input of nutrients to soil (Table S1, Supporting Information). Lawes never used these and similar results to suggest that fertilizers were better than FYM. Rather, he realized that no farmer would have this amount of FYM to apply to each field each year and that fertilizers, when used judiciously, could maintain and increase food production to help feed the rapidly increasing urban population.

Fertilizers and FYM continued to give very similar yields of wheat, barley (*Hordeum vulgare* L.), mangolds and sugar beet (*Beta vulgaris* var*. altissima*) in the 1960s–1970s (Table S2, Supporting Information) even though there was now 2.5 times more organic matter in the soil where 35 t ha^−1^ FYM had been applied annually for more than 100 years. This led some to conclude that soil organic matter (SOM) was not essential to achieve good yields. However, as crop cultivars with increased yield potential have been introduced, yields in many Rothamsted long‐term experiments are now larger on soils with more SOM (Johnston *et al*., 2009), not least because SOM improves soil structure. This is especially important for spring‐sown crops, which need to develop a good root system rapidly to take up nutrients and water quickly to achieve their yield potential.

## The agricultural value of long‐term experiments: testing sustainability

Our only realistic assessment of the sustainability of an agricultural system is the yields of the crops grown, and for those yields to be maintained or increased there must be a match between the crop, soil, climate and management of the system. Assuming the first three conditions are met, management becomes important.

**Figure 1:**
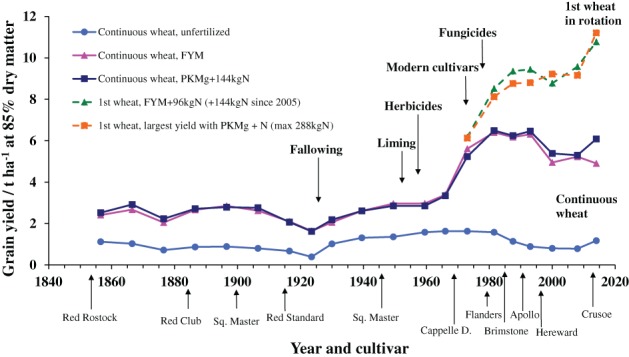
Mean long‐term yields of winter wheat grain, 1852–2016, showing selected treatments, important changes in management and cultivars grown. Broadbalk Winter Wheat experiment, Rothamsted.

**Figure 2:**
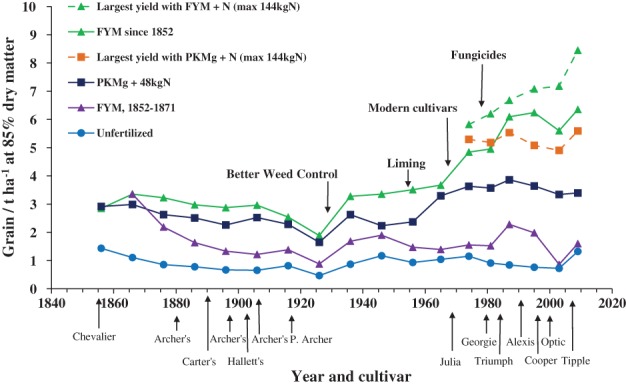
Mean long‐term yields of spring barley grain, 1852–2015, showing selected treatments, important changes in management and cultivars grown. Hoosfield Spring Barley experiment, Rothamsted.

Figure 1 shows the yields of winter wheat on Broadbalk and Figure 2 the yields of spring barley on Hoosfield, and the management changes that have helped to maintain and increase yields in both experiments. In many experiments, there are defining moments or decisions that have a major influence on their importance; for Broadbalk, there have been several. In the 1950s, to counteract the acidifying effect of ammonium sulphate on the plots on which it was tested, appropriate amounts of chalk were applied to maintain soil pH above 7. To control weeds by cultivation, the experiment was divided into five sections in 1926 so that each could be fallowed in turn while wheat was grown on the other four sections. When weed‐killers became available in the 1950s they were tested first on a limited area, and when shown to have no detrimental effect on yield they were used on all but one half‐section from 1964. Once soil acidity and weeds were being managed, there was a need to consider how best to maintain the relevance of the experiment (and others) to mainstream farming.

Following a comprehensive review, major modifications were made from 1968. Short‐straw cultivars, which were being grown widely by this time, were introduced and this allowed larger amounts of N to be tested, especially when growth regulators were used to help prevent lodging. The form in which N was applied was changed also from ammonium sulphate to calcium ammonium nitrate (it was changed again in 1986 to ammonium nitrate). Each of the existing five sections were halved to make 10 sections, such that continuous wheat could be compared with a first wheat grown following 2 years without a cereal crop susceptible to take all (*Gaeumannomyces graminis*). Figure 1 shows that the yields of the first wheat after a 2‐year break are larger than those of continuous wheat, suggesting that farmers should grow a first wheat as frequently as possible. There was also a benefit from controlling foliar pathogens with fungicides when required. Some of these management changes (liming, and fallowing to control weeds) were essential to ensure that the experiment continued. Other changes were made for good scientific reasons so that yields could be maintained or increased. The largest yields can now exceed 12 t ha^−1^ with FYM plus extra N in the spring or with P, K, Mg plus 240 or 288 kg N ha^−1^. However, the initial aim, which was to identify the nutrient requirements of winter wheat, still remains. As on Broadbalk, yields of spring barley on Hoosfield have increased with the introduction of cultivars with a greater yield potential, applications of larger amounts of N and better weed control (Figure 2).

### Soil acidity

Management issues include the control of soil acidity (unless the effects of acidity are to be tested), the maintenance of adequate amounts of plant‐available nutrients and soil structure. Lawes & Gilbert made little or no reference to soil acidity; expressing acidity on a pH scale was some decades off. In the mid‐1800s (based on analysis of archived soils) most arable fields at Rothamsted had a pH_water_ well above 7 following the application of large amounts of chalk, probably 100 years earlier. In the Agdell rotation experiment on a silty clay loam soil at Rothamsted, it took 80 years on plots given NPK fertilizers (N as ammonium sulphate and rape cake) for the yield of turnips to decline to near that on the unfertilized plot. This was not a direct effect of acidity, but was mainly caused by the build‐up of the fungus *Plasmodiophora infestans*, which causes ‘finger and toe’ in turnips in acid soil (Powlson & Johnston, 1994). On the sandy loam at Woburn, with an initial pH_water_ *c*.6 and little CaCO_3_ in the soil, acidity developed quickly where 46 kg N ha^−1^ as ammonium sulphate was applied annually from 1877 to1926, and yields of spring barley declined from 2.57 to 0.30 t ha^−1^ (Johnston & Chater, 1975). Although the adverse effect of acidity on yield either directly or in conjunction with pests or diseases and its amelioration are now well understood, suitable liming materials to correct acidity might not always be readily available.

### Availability of soil phosphorus

Single superphosphate was tested in all the long‐term Rothamsted experiments, at 33–35 kg P ha^−1^. In laboratory experiments, Way (1850) showed that water‐soluble phosphate was retained in soil, and later Lawes's field experiments showed little residual benefit to crops from the large amounts of P applied for many years. Subsequently, mainly laboratory studies appeared to confirm the irreversible retention or fixation of P in soil in forms not available to plants, but there were some indications that this was not generally correct (see discussion in Syers *et al*., 2008). Irreversible P fixation was questioned further because, after the application of basal N since 1949, yields of spring barley in the Exhaustion Land experiment at Rothamsted were larger on soils that had received P from 1856 to 1901 but none since, than on soils without P since 1856 (Table 1), (Johnston & Poulton, 1977). This started much research on the plant availability of P residues, their measurement by soil analysis, and the extent to which they should be accumulated in soil (Johnston *et al*., 2013; Poulton *et al*., 2013). The results suggested that soils growing arable crops and grass in England, Wales and Northern Ireland could be maintained at about 20 mg kg^−1^ Olsen P without major risk of loss of yield from lack of P.

**Table 1:** Mean yields of spring barely grain, Exhaustion Land, Rothamsted

Period	Cultivar	N applied / kg ha^−1^	Plot and treatment		
			1, 2, 5, 6 No P or K since 1856 Grain / t ha^−1^	7, 8 Residues from PK applied 1856–1901	3, 4 Residues from FYM applied 1876–1901
1949–1963	Plumage Archer	63	1.8	2.9	3.2
1970–1975	Julia	88	1.8	4.2	4.8
1980–1983	Georgie	96	1.1	2.7	3.8

### Agronomic importance of soil organic matter

**Figure 3:**
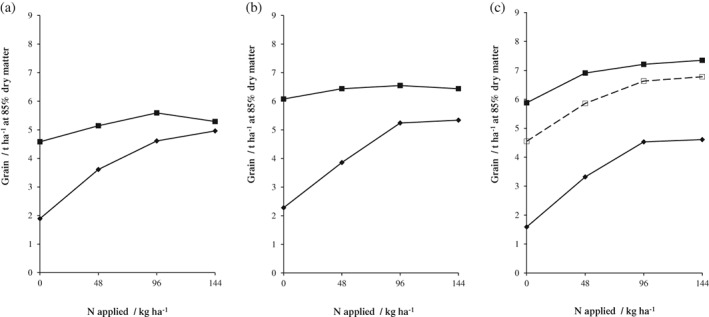
Mean yields of spring barley grain for selected treatments: (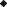) P K Mg; (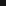) farmyard manure (FYM) since 1852; (□) FYM since 2001. (a) cultivar Julia, 1976–1979, (b) cultivar Triumph 1984–1991 and (c) cultivar Optic 2004–2007. Hoosfield Spring Barley experiment, Rothamsted.

For most crops to achieve their yield potential they need to grow in soil with a good structure to access nutrients and water. Two examples are: (i) in experiments on the availability of soil P, the critical concentration of Olsen P was smaller on soils with more SOM (Table 2) and (ii) Figure 3 shows the yields of three cultivars of spring barley grown in the Hoosfield barley experiment in different periods after 1968 when four rates of N were tested on all plots. Where PK fertilizers (35 kg P ha^−1^ and 90 kg K ha^−1^) or FYM (35 t ha^−1^) had been applied annually since 1852, the soil with FYM contained 2.5 times more SOM than that with fertilizers. Average grain yields of *cv*. Julia (1976–1979) with 144 kg N ha^−1^ were the same on both soils; the extra SOM did not appear to be important. In 1984–1991, the yield of *cv*. Triumph, with a larger yield potential, was less on the fertilizer plot than on the FYM plot, and with some additional fertilizer N yields increased further on the FYM plot. Changing to *cv*. Optic in 2004–2007 appreciably increased yields on the FYM plots with little further increase on the fertilizer plots. For these three cultivars with increasing yield potential, growing them on soil with more SOM increased yields. Interestingly, even with the better cultivars and applying 144 kg N ha^−1^, the yield on the fertilizer plots has ranged from only 4.9 to 5.5 t ha^−1^, whereas on the FYM plus N plots yields have been almost 8 t ha^−1^.

**Table 2:** Effect of soil organic matter on the critical level of Olsen P for three arable crops grown on a silty clay loam soil, Agdell, Rothamsted

Crop	Soil organic carbon / %	Yield at 95% of the asymptote / t ha^−1^	Olsen P associated with the 95% yield /mg kg^−1^	Variance accounted for / %
Spring barley grain	2.4	5.26	16	83
	1.5	4.69	45	46
Potato tubers	2.4	47.1	17	89
	1.5	46.5	61	72
Sugar, from	2.4	6.92	18	87
Sugar beet	1.5	6.91	32	61

**Figure 4:**
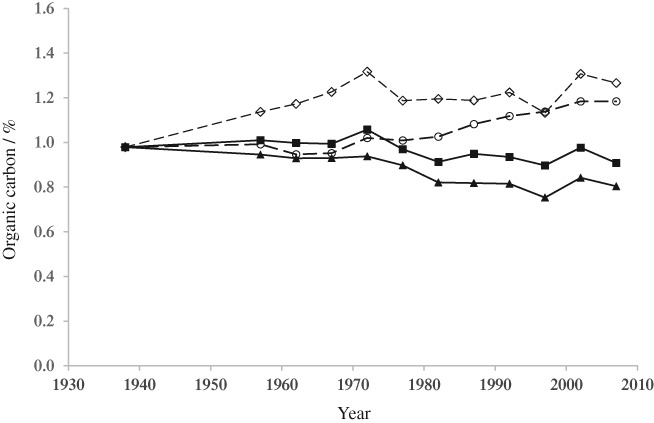
Percentage of organic C in soil to 25‐cm depth for selected treatments: (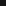) AB, all‐arable rotation; (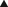) AF, arable rotation with root crops or fallows; (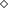) LN3, 3‐year grass leys + N followed by two arable crops; (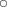) LC3, 3‐year grass + clover leys followed by two arable crops. Data are the mean of soils with or without FYM; each point is the mean of data from each of the five blocks. Ley–arable experiment, Woburn. (From Johnston et al., 2017.)

In temperate climates, the amount of SOM changes slowly, often over many decades, and data from the Rothamsted experiments show that it eventually reaches an equilibrium level depending on the soil and cropping (Johnston *et al*., 2009). Most recently, the change in SOM over a period of 70 years has been measured in four 5‐year crop rotations, two with all‐arable crops and two with a 3‐year ley and two arable crops. With the ley rotations, % OC increased to, and was maintained at, a higher level than in the all‐arable rotations (Figure 4). When the increase is expressed as t ha^−1^ the data showed that only *c*. 5% of the added OC was retained in the soil; there was little indication that a change from all‐arable to ley–arable cropping would fix large amounts of atmospheric CO_2_ in the extra SOM (Johnston et al., 2017).

## Ecological research and long‐term experiments

The Park Grass experiment, started in 1856, was the last major field experiment started by Lawes and Gilbert. The original aim was to measure the yield of hay and later regrowth with FYM and N, P and K applied singly and in combination as fertilizers, with N applied as either ammonium sulphate or sodium nitrate. Having observed that arable crops belonging to different plant families responded to N, P and K in somewhat different ways, and being aware that species in the same families occurred in the permanent sward on Park field, Lawes decided to see if they responded in the same way when in a permanent sward. The unexpected result was that after a few years, the experiment looked more like one on seed mixtures than one on fertilizers, and botanical surveys were started to record the effect on species diversity (Lawes & Gilbert, 1863). Although yields are still measured, Park Grass is now best known as the most long‐term ecological study.

Unlike the arable fields at Rothamsted, permanent pasture fields, like Park field, never received large amounts of chalk so that in 1856 the soil was about pH_water_ 5.5. Lawes and Gilbert made some simple tests of liming between 1881 and 1896, but without any effect on hay yields. In 1903, a more rigorous test of liming was started by halving most plots and applying chalk at 4 t ha^−1^ CaCO_3_ to one half‐plot every fourth year. Gradually a difference in soil pH developed between the half‐plots, together with a difference in the species composition of the sward (Warren & Johnston, 1964).

**Figure 5:**
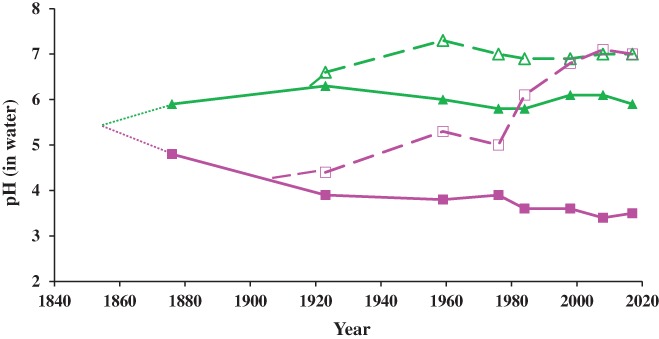
Soil pH_(water)_ in topsoil, 0–23 cm, of plots receiving P, K, Na, Mg and different forms of N fertilizer. Treatments are: (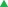) 96 kg N ha^−1^ year^−1^ as sodium nitrate, no lime; (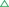) 96 kg N ha^−1^ year^−1^ as sodium nitrate, with 4 t CaCO_3_ ha^−1^ every fourth year 1920–1964, with sufficient CaCO_3_ to maintain pH 7 since 1965; (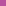) 96 kg ha^−1^ year^−1^ as ammonium sulphate, no lime; (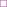) 96 kg ha^−1^ year^−1^ as ammonium sulphate, with 4 t CaCO_3_ ha^−1^ every fourth year 1903–1964, with sufficient CaCO_3_ to increase and then maintain soil at pH 7 since 1965 (see text for details). Park Grass experiment, Rothamsted.

Although the half‐plots had chalk every fourth year, by the early 1960s there was very little overlap in the pH of the top 23 cm of mineral soil between plots receiving ammonium sulphate and sodium nitrate (Figure 5). Thus, it was not easy to compare the effects of ammonium and nitrate on species composition and Warren & Johnston (1964) suggested that a wider range of soil pH was required to separate more accurately the effects of nutrients and soil acidity on the species composition of the sward. This was achieved by dividing most of the half‐plots to make four subplots, a, b, c and d, on most of the original plots. The aim was to establish and then maintain soil (0–23 cm) with pH values of 7, 6 and 5, on subplots a, b and c, respectively, by the addition of chalk. Interestingly, the pH of the mineral soil changed little on those subplots with a surface mat of organic material until the pH of the mat was raised, which encouraged microbial activity to speed its decomposition (Johnston, 1972). Subplot d does not receive chalk and has the lowest pH resulting from the anthropogenic and natural inputs; these values now range from pH 5.0 on the unmanured plots to 3.4 where the largest amount of ammonium sulphate is applied. Now that soil pH is controlled more precisely, the effect of nutrient availability and soil acidity on species composition, together with change in species over time, have been recorded (Crawley et al., 2005; Silvertown et al., 2006) and theories of resource competition both above and below ground have been developed (Tilman, 1982; Grime *et al*., 1988).

One intriguing feature of Park Grass is the evolutionary changes in *Anthoxanthum odoratum* L., which has evolved to be both morphologically and physiologically adapted to the soil conditions and nutrient supply in some of the plots on which it grows (Snaydon, 1970; Snaydon & Davis, 1976).

Atmospheric N pollution and addition of N fertilizers, especially on the more acid soils, had caused a decline in species numbers by the 1960s on some plots (Crawley et al., 2005). The recent decrease in atmospheric N inputs and ceasing to apply fertilizer N to a part of some plots has led to an increase in species number on these plots (Storkey *et al*., 2015).

The other ecological study of current relevance is the natural regeneration of woodland on old arable soils that has occurred on Broadbalk Wilderness since 1882 and Geescroft Wilderness since 1884, and the increase in SOM in these soils (Poulton *et al*., 2003; Johnston *et al*., 2009). Of interest, has been the greater acidification of soil in Geescroft than in the unmanured plot on Park Grass because the trees trapped a greater proportion of the atmospheric acidifying inputs than the grass. This has then leached into the soil, resulting in the mobilization of heavy metals (Blake & Goulding, 2002).

## Long‐term experiments and environmental issues

Inorganic and organic pollutants are a threat to the sustainability of any agricultural system. Linking their concentrations in archived and current soil samples shows how they have changed in response to natural and anthropogenic inputs and how their concentrations have changed in the crops grown on the soils, as shown for cadmium (Cd), lead (Pb) and organic pollutants in the Supporting Information.

### The ‘4 per 1000’ initiative

The most recent use of data from the long‐term experiments is in relation to this initiative. In response to concern about the role of atmospheric CO_2_ in global warming, the French Ministry of Agriculture in 2015 launched the ‘4 per 1000 initiative: Soils for Food Security and Climate’ at the Paris conference of the United Nations Framework Convention on Climate Change (UNFCC) (http//4p1000.org/understand). The suggested aim is to increase the amount of SOC to a depth of 40 cm by 4‰ (0.4%) per year for 20 years and thus halt the increase in atmospheric CO_2_. We have prepared an evidence‐based assessment for publication of those situations in practical agriculture where, with a change in cropping or management, the 4‰ target might be achieved using data from 16 long‐term Rothamsted experiments on three soil types with more than 110 treatment comparisons.

## Statistics, curve fitting and modelling

Lawes and Gilbert knew every detail of their experiments and presented and discussed so many of them in their papers that important conclusions are often difficult to find. R.A. Fisher was appointed as a statistician in 1919 to “examine our data and elicit further information that we had missed …. and advise on whether there was much that required proper statistical examination” (Russell, 1966). Fisher, working mainly on the wheat yields on Broadbalk and the hay yields on Park Grass, laid the foundations on which modern statistical science is based. He made major contributions to fitting curves to data, refining notions of regression and analysis of variance, and the use of multifactor or factorial experiments, and consequently the notion of statistical significance. More recently, the statistical software package GenStat^®^ has been developed. Rothamsted statisticians have also helped us develop the following ideas.

### Separating the effect on yield of nitrogen and other factors

**Figure 6:**
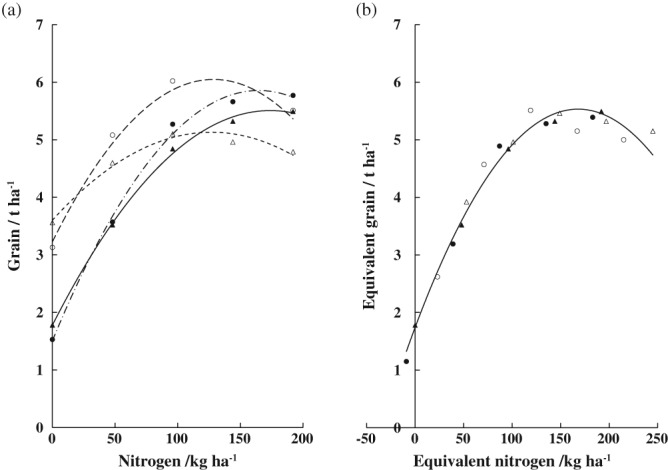
Relation between nitrogen applied and mean yield of grain (t ha^−1^) grown in four rotations: (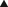) continuous wheat; (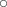) wheat after a 2‐year break; (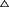) first wheat after a 1‐year fallow; (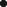) second wheat after a 1‐year fallow. (a) Individual fitted N response curves and (b) fitted N response curves brought into coincidence by vertical and horizontal shifts. Broadbalk Winter Wheat experiment, 1970–1978, Rothamsted. (Adapted from Dyke et al., 1983.)

From 1970 to 1978, wheat on Broadbalk was grown in four scenarios: continuously, as a first wheat after a 2‐year break, and as a first and second wheat after a 1‐year fallow, each with five levels of N (Dyke *et al*., 1983). An exponential plus linear response curve was fitted to the individual N‐response data, and the individual curves for each scenario were brought into coincidence (Figure 6a). The linear term in the curve fitting model gave a maximum yield, and by diagonal shifts to superimpose the maximum yields, the four curves could be brought into coincidence (Figure 6b); the combined curve accounted for 97.3% of the variance. In this example the maximum yields were shifted to the response curve for the wheat grown continuously. In doing this for the other three scenarios, their corresponding *x*‐axis and *y*‐axis were also effectively shifted (see Figure 4 in Dyke *et al*. 1983). Thus, in Figure 6(b), the *x*‐axis is best shown as an equivalent N‐scale and the *y*‐axis as an equivalent yield scale. The diagonal shift has a horizontal component related to available N in the spring, and the vertical component is related to differences in potential yield, in this case probably related to the incidence of take all. Compared with wheat grown continuously, the diagonal shifts in the graphs represented: (i) a benefit of 53 kg ha^−1^ available N but 0.36 t ha^−1^ less grain for the first wheat after a 1‐year fallow, (ii) 9 kg ha^−1^ less available N but 0.38 t ha^−1^ more grain for the second wheat after the fallow and (iii) 23 kg ha^−1^ more available N and an extra 0.51 t ha^−1^ grain for the wheat after field beans (*Vicia faba*) (Dyke *et al*., 1983). Similar curve shifting has been used to help define the benefits of FYM in the Broadbalk experiment from 1979 to 1984 (Johnston, 1987) and of SOM derived from growing grass–clover leys (Johnston *et al*., 1994). Two further examples of curve shifting related to microbial decomposition of SOM and decline in plant‐available P in soil are in the Supporting Information (Figures S2 and S3).

### The Rothamsted carbon model

**Figure 7:**
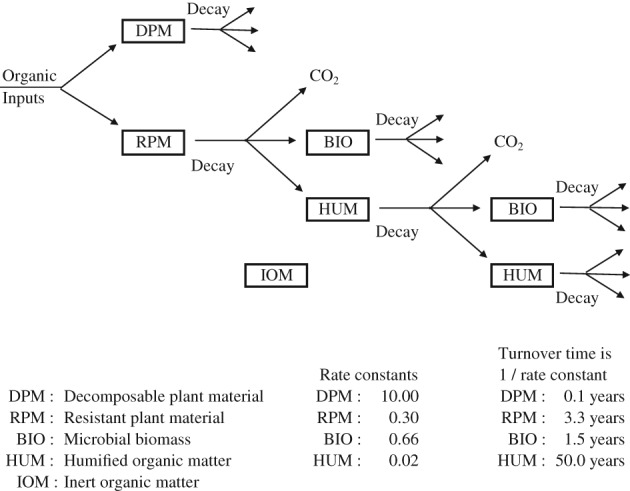
Schematic diagram showing the flow of carbon through the Rothamsted turnover model (RothC‐26.3) with rate constants and turnover times for the different carbon pools. Redrawn from Jenkinson et al. (1994). Plant material that enters the soil is partitioned into two input compartments: decomposable plant material (DPM) and resistant plant material (RPM). Both DPM and RPM are decomposed in the soil by first‐order processes to CO_2_, which is lost from the system, and to microbial biomass (BIO) and humified organic matter (HUM), which are both retained in the soil. Both BIO and HUM are decomposed further to give more CO_2_, biomass and humified matter. The soil is also assumed to contain a small (c. 10%) amount of inert organic material (IOM), which is inert to microbial decomposition (at least in the short term).

**Figure 8:**
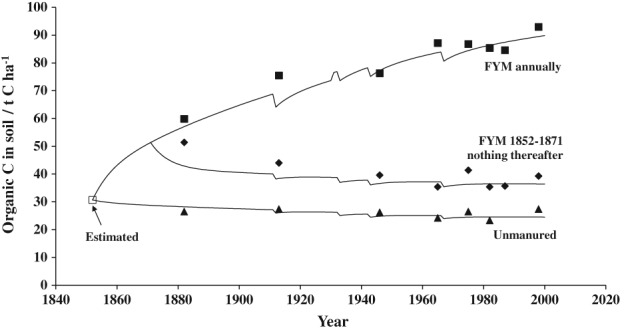
Organic carbon (t ha^−1^) in the top 23 cm from three plots growing spring barley. Treatments are: (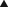) unmanured; (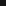) farmyard manure (FYM), 35 t ha^−1^ annually; (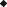) FYM 35 t ha^−1^ annually 1852–1871, none since. The data points are adjusted for changes in soil bulk density and the solid lines are the model output. The FYM, ploughed‐in in February 1852–1930 and in late autumn after 1932, was assumed to contain no biomass but decomposable plant material (DPM), resistant plant material (RPM) and humified organic matter (HUM) in the proportions 0.49, 0.49 and 0.02, respectively. The incoming plant residues were assumed to have DPM and RPM in the proportion 0.59 and 0.41, respectively. The IOM for these treatments contained 2.7 t C ha^−1^. See Figure 7 for explanation of DPM and so on. To obtain a (modelled) value of carbon in the soil at the start of the experiment a plant debris input of 1.69 t C ha^−1^ was used. Thereafter, the annual C inputs, t ha^−1^, were: unmanured plot, 1.28 (from plant debris); FYM plot, 2.8 (from plant debris) plus 3.0 (from FYM); FYM residues plot as FYM plot 1852–1871 then 2.0 (from plant debris) after 1872. Hoosfield Spring Barley experiment, Rothamsted. (From Johnston et al., 2009.)

With the large amount of data on organic C in the top 23 cm of the soil in the Rothamsted experiments, a model was developed to describe the changes. The model, ROTHC‐26.3 (Jenkinson, 1990; Jenkinson *et al*., 1994), is a five‐compartment model (Figure 7). The fit of the model to the observed changes in C, in t ha^−1^, for three treatments in the Hoosfield Barley experiment is good (Figure 8), except for the first few years when SOM was declining in the plot where FYM applications stopped after the first 20 years of the experiment. Figure 8 is a true test of the model because no data from the experiment were used to set the model parameters and no adjustments were made to improve the fit. Other examples of using the model are in Johnston *et al*. (2009) and there is another more recent example from the Woburn Ley–arable experiment (Johnston *et al*., 2017).

## Ancillary experiments and use of archived samples

Examples of two ancillary experiments sited within existing experiments or using archived material follow, with two further examples in the Supporting Information (Figure S1).

### The use of stable isotopes

The long‐term experiments, where SOM is in equilibrium in many treatments, have been used for detailed research on N cycling using the stable isotope, ^15^N, applied to unconfined microplots within Broadbalk, Hoosfield and Park Grass. Results showed that, on average, *c*. 50% of the fertilizer applied was taken up by the crop, *c*. 25% remained in the soil and *c*. 25% was lost by leaching or denitrification (Powlson *et al*., 1986; Glendining *et al*., 1997; Jenkinson *et al*., 2004). Importantly, the results showed that *c*. 1% (range 0–4%) only of the fertilizer N applied in spring was present as inorganic N in the soil in autumn, and thus at risk of leaching (Macdonald *et al*., 1989).

### Selenium

The intake of selenium (Se) by the UK population has declined substantially since the 1970s and this is a cause for concern because Se is an essential element for human health. (Oliver & Gregory, 2015). Bread is a source of Se and the concentrations in the wheat grain grown on Broadbalk since the 1840s on the unmanured, fertilizer and FYM plots have been measured to see whether there has been a marked decrease in Se concentrations following the dramatic increase in grain yield (Figure 1) since the 1960s. No significant trends in grain Se concentrations have been detected in the fertilizer and FYM plots, which suggests that this does not explain the declining levels in the human diet. In general, on the unmanured plots Se concentrations were smaller between 1920 and 1970 than before or after these years, a pattern that mirrored the temporal pattern of SO_2_ emissions, suggesting that large S inputs decrease Se concentrations in grain. Soil Se concentrations have tended to increase on all plots since the 1840s (Fan *et al*., 2008).

## Experiments that failed, were stopped or were extensively modified

Not all long‐term experiments at Rothamsted have survived; some were stopped when there was no longer scientific interest or specific funding. Here, we discuss some that have been stopped completely or have been extensively modified; other examples are in the Supporting Information.

### Leguminous grain and forage crops

Both crops had an important role in early 19^th^‐century agriculture in the UK as a source of N, especially for wheat for bread that followed beans or clover in the Norfolk four‐course arable rotation. From 1847, field beans were grown each year on Geescroft, and from 1849 attempts were made to grow clover continuously on a part of Hoosfield. Neither crop could be grown successfully year after year, as wheat and barley could in their long‐term experiments. Lawes and Gilbert could not explain this, although they continued to seek an explanation by starting new experiments on clover for some years in different fields. The experiment on Hoosfield was stopped in the early 1900s. As mentioned previously, part of the Geescroft experiment was fenced off and left untended in 1886 and trees have regenerated naturally (Poulton *et al*., 2003).

The original Agdell rotation experiment, 1848–1951, failed because of soil acidity. After soil pH was raised by applying chalk, half of each plot was sown to grass in 1958, whereas the other half remained in arable crops. From 1958 to 1970, the residual value of the accumulated P and K residues available for the arable crops and grass was measured (Johnston & Penny, 1972). In 1964, the plots had been divided further to establish four levels of Olsen P and four levels of exchangeable K, as on the Exhaustion Land (see below), with the unique opportunity to do this at two levels of SOM in this one experiment after growing grass for 12 years. The critical level of plant‐available Olsen P for three arable crops at two levels of SOM is shown in Table 2 (Johnston & Poulton, 2011). By 1973, successive modifications had resulted in each of the six original large plots (each 24 m × 58 m) being divided into 64 sub‐plots (each 6 m × 6 m). Although small, the plots could be ploughed, cultivated and drilled or planted with conventional farm machinery, but harvesting was by hand. The experiment was stopped in the late 1980s.

### Assessing the nutrient availability of soil P and K reserves

**Figure 9:**
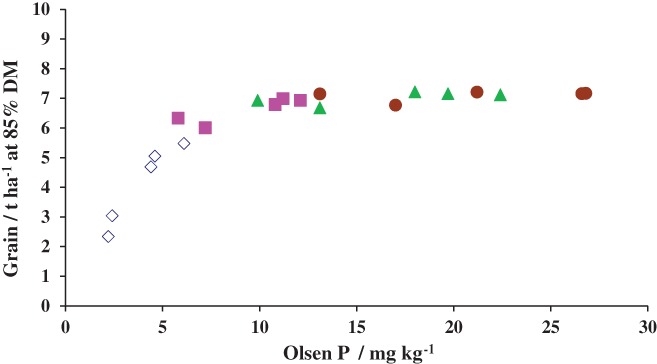
Response of spring barley to Olsen P in topsoil, 0–23 cm, after 3 years of fresh fertilizer P applications following 85 years when no P was applied. Treatments are: (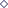) no fresh P; (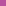) 44 kg P ha^−1^ year^−1^; (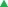) 87 kg P ha^−1^ year^−1^; (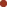) 131 kg P ha^−1^ year^−1^. Exhaustion Land experiment, Rothamsted.

The history of the Exhaustion Land shows how this Rothamsted experiment has been modified to answer ‘new’ questions. The yields of winter wheat (1856–1874) and then potatoes (1876–1901) were measured on 10 plots testing combinations of N, P and K as fertilizers and FYM. The site was cropped without further additions of fertilizers until 1941, when N was applied to all plots, and since 1949 yields on plots with combined P and K residues from fertilizers or FYM have been larger than on those without (Table 1) (Johnston & Poulton, 1977). Half of the plots were assigned to test plant‐available P in 1985 in order to respond to emerging questions: How much plant‐available P should there be in the soil? How quickly could the decline in available P be reversed? Each of the five main plots was divided into four and a range of Olsen P established on plots given basal K together with N, and the response, first of barley and then of winter wheat, to Olsen P was measured (Poulton *et al*., 2013). Figure 9 shows the response of spring barley to Olsen P in 1988. The response of wheat to exchangeable K is measured on the other half of the experiment.

## Essential requirements for long‐term agricultural field experiments

For most crops that are grown in the field, the suitability and fertility of the soil is reflected in the yields of the crops grown in the absence of weeds, pests and diseases. On a plot basis, and in any one year, the crop reflects the conditions in the soil better than a limited number of soil samples can. But, poor yields are best explained by taking representative soil samples for chemical analysis and making a physical examination of the conditions within the soil profile that might affect yield. Long‐term experiments are a field‐based resource and together with crop and soil samples provide material for research. For example, current and archived soil samples have been used to develop the Rothamsted carbon turnover model (Jenkinson, 1990; Jenkinson *et al*., 1994) and provide a better understanding of the behaviour of soil P (Syers *et al*., 2008). Analysis of crop and soil samples from the long‐term K experiment has shown how N efficiency depends on the availability of soil K to the plant (Milford & Johnston, 2007).

### Treatments and management

Deciding on treatments and experimental design will depend on the questions asked and could involve biological, chemical and physical factors, singly and in combination. Consequently, a multidisciplinary group of researchers will need to form a management group with overall responsibility for the experiment and publication of the results. But, day‐to‐day management of the field operations should be the responsibility of a well‐qualified, experienced person with appropriate agronomic skills. The management group will need to decide if and what modifications are required to answer any new questions that may arise. For example, changes in the Woburn Ley–arable experiment were made after careful consideration to ensure its continuity and achieve its original objective of comparing the productivity of 5‐year rotations, two with all‐arable cropping, two with a 3‐year ley followed by the same two arable test crops. In this experiment, valuable additional information could be obtained, which should be the aim with all long‐term experiments. In this case, it is related to the equilibrium level of SOM in the different rotations and the small amount of carbon fixed from atmospheric CO_2_ (Johnston *et al*., 2017). A more detailed account of the changes in the Woburn Ley–arable experiment is given in the Supporting Information (Table S3).

The choice of the site is crucial to the success of taking account of soil texture and nutrient status, topography and ease of access. Plot size is important and on reasonably uniform soil large plots would be used to make visual comparison of the effects of the treatments easy, and they can be divided subsequently into smaller plots to test additional factors of interest.

All input materials such as fertilizers and manures, and all outputs such as grain, straw and potato tubers, should be recorded and sampled for immediate or future analysis. Crop yields should be measured; monitoring these for unexplained variation from the long‐term means is the role of the management group, and samples will be analysed for major and micronutrients of immediate interest. Many soil properties that are likely to affect the continuity of an experiment may change only slowly; therefore, soil samples can be taken periodically. They should always be taken to the same depth, at the same time of the year and at the same point in the crop rotation if applicable and analysed for biological, chemical and physical properties of immediate interest. A subsample of all the inputs and outputs should be archived. Occasionally, soil samples should be taken to a greater depth and bulk density determined. Local meteorological data should be recorded either manually or with an automatic recording system. Recording necessary financial information is important if treatment effects are to be costed. Whether determined because of the questions being asked of an experiment or to explain variations in annual yields, the incidence of pests, diseases and weeds should be monitored if possible.

### Site tenure and costing

Long‐term experiments are costly but, as shown here, they should and can contribute to more than one objective. The two most important requirements for all long‐term agricultural field experiments are security of tenure of the site and committed long‐term funding. Rothamsted itself is perhaps unique in that in 1889 Lawes provided financial support for the continuation of the field experiments by endowing the Lawes Agricultural Trust (LAT) with £100 000, and establishing The Lawes Agricultural Trust Committee (LATC) to give scientific support. In 1934 the LAT purchased the whole of the estate, ensuring the tenure of the Rothamsted farm and experiments. Research at Woburn was started by The Royal Agricultural Society of England in 1876 on a farm at Woburn leased from the Woburn Settled Estates. The lease was transferred to the LATC in 1926. Where land is leased, care is required to ensure the continuation of the lease.

Initially, the experiments and ancillary services were financed wholly by Lawes, and after the endowment to the LAT in 1889 it was expected to meet the costs. The first public money came to Rothamsted in 1911 from the Development Commission and then government grants were made annually, initially from the Agricultural Research Council. Following the reduction and withdrawal of direct government support, Rothamsted has, like many similar organizations, to seek funding from a wide range of grant‐giving bodies. Currently, the long‐term experiments are maintained as a National Capability by the Biotechnology and Biological Sciences Research Council of the UK. In developed countries, the present affluence and ability to buy food should not lead to a false sense of everlasting food security and a holistic approach to experiments designed to ensure food security is essential.

## Conclusions

The long‐term experiments at Rothamsted were probably never intended to be long term. Lawes and Gilbert would probably have stopped their field experiments after a few years if there had not been a long‐continued argument with Liebig about the source of N for crops. By the time this controversy ended they were aware that farmers were taking an increasing interest in the results. They themselves saw increasing scientific interest, and they published their last paper in 1900 and made major changes to the experiments as late as the 1880s. It was in 1882 that Lawes commented that the value of the experiments had increased with time, together with that of the archive of crops and soils. These, and the value of the detailed records they kept, continue to increase. Appropriate changes to the experiments and their management make them ever‐more important in relation to the sustainability of food production systems and environmental issues related to agriculture. Current data provide information of immediate use to farmers and can also be used to inform government policy in relation to land use and agriculture.

## Supporting information


**Supporting Information** Background to the start of the Rothamsted long‐term experiments.
**Table S1.** Important early results from the Broadbalk Wheat experiment.
**Table S2.** Yields of winter wheat and spring barley grain and roots of mangolds and sugar beet at Rothamsted.
Long‐term experiments and environmental issues.
**Figure S1.** Concentrations of lead in herbage, 1956–1988, for plots receiving NPK fertilizers. Reference Plots, Rothamsted. (From Jones *et al*., 1991.)
Curve shifting
**Figure S2.** Decline in organic C in the top 23 cm of soil. Symbols denote previous inputs of FYM, sewage sludge, FYM compost and sludge compost. After treatments stopped, soils were sampled over the next 5–12 years. The data could be shifted horizontally to fit a common exponential model. Market Garden experiment, Woburn. (Adapted from Johnston *et al.*, 1989.)
**Figure S3.** Change in Olsen P on a sandy clay loam given no P for 14 years: (a) data for eight individual treatments and (b) an exponential decay curve fitted to the data for the eight treatments once the curves have been bought into coincidence by a series of horizontal shifts. Different symbols denote treatments that started at different concentrations of Olsen P. Rotation II experiment, Saxmundham. (From Johnston *et al*., 2016.)
Ancillary experiments.
Some of the challenges faced in maintaining the Woburn Ley–arable experiment.
**Table S3.** Initial treatment and test crops, Woburn Ley–arable experiment.
Experiments that were stopped: Woburn permanent wheat and barley experiments and the rotation experiments.
**Photographs** Photographs of some of the long‐term experiments at Rothamsted and of the sample archive.
