# Supplementary material for: The importance of long‐term experiments in agriculture: their management to ensure continued crop production and soil fertility; the Rothamsted experience
Source: Eur J Soil Sci. 2018 Jan 18;69(1):113–25. doi: 10.1111/ejss.12521 (PMC5832307; doi:10.1111/ejss.12521)
Supplement: Supplementary file 1 — Supporting Information Background to the start of the Rothamsted long‐term experiments. Table S1. Important early results from the Broadbalk Wheat experiment. Table S2. Yields of winter wheat and spring barley grain and roots of mangolds and sugar beet at Rothamsted. Long‐term experiments and environmental issues. Figure S1. Concentrations of lead in herbage, 1956–1988, for plots receiving NPK fertilizers. Reference Plots, Rothamsted. (From Jones et al., 1991.) Curve shifting Figure S2. Decline in organic C in the top 23 cm of soil. Symbols denote previous inputs of FYM, sewage sludge, FYM compost and sludge compost. After treatments stopped, soils were sampled over the next 5–12 years. The data could be shifted horizontally to fit a common exponential model. Market Garden experiment, Woburn. (Adapted from Johnston et al., 1989.) Figure S3. Change in Olsen P on a sandy clay loam given no P for 14 years: (a) data for eight individual treatments and (b) an exponential decay curve fitted to the data for the eight treatments once the curves have been bought into coincidence by a series of horizontal shifts. Different symbols denote treatments that started at different concentrations of Olsen P. Rotation II experiment, Saxmundham. (From Johnston et al., 2016.) Ancillary experiments. Some of the challenges faced in maintaining the Woburn Ley–arable experiment. Table S3. Initial treatment and test crops, Woburn Ley–arable experiment. Experiments that were stopped: Woburn permanent wheat and barley experiments and the rotation experiments. Photographs Photographs of some of the long‐term experiments at Rothamsted and of the sample archive. [file EJSS-69-113-s001.docx]

**The importance of long-term experiments in agriculture—their management to ensure continued crop production and soil fertility; the Rothamsted experience**

A.E. JOHNSTON & P.R. POULTON

**Supporting Information**

**Background to the start of the Rothamsted long-term experiments**

In the 1830s, Professor C. G. B. Daubeny, Professor of Rural Economy, was doing experiments in the Botanic Gardens at the University of Oxford on the effect of a plant on the growth of one that followed, and advocating the need for more experiments on the growth of agricultural crops generally. Lawes left Oxford in 1834 to manage the small Rothamsted agricultural estate, but while there he had been greatly influenced by Professor Daubeny as seen later. At that time applying crushed bones to some soils greatly increased yields such that in 1837 domestic use was 27 000 t while imports were 46 000 t valued at £255 000. Justus von Liebig accused the British of raiding the battlefields of Europe to satisfy this demand! But crushed bones had no effect on Rothamsted farm and intrigued by the lack of benefit Lawes, in 1837 and 1838, did some experiments in pots and small plots on his farm testing various water-soluble ammonium salts made by neutralizing ammoniacal liquor from town gas production with the appropriate acid. Applied to supply the same weight of nitrogen (N) they were tested on cabbages used as animal feed, and the yields of 25 cabbages varied greatly. With ammonium phosphate, the yield was 28 kg, with ammonium sulphate 18 kg, with ammonium chloride 13 kg, with ammonium nitrate 10 kg and with ammonium carbonate 5 kg (Lawes, 1842, 1843). The phosphate was water-soluble (unlike that in bones) and the soil was deficient in phosphorus (P) demonstrating the need for applied P to be in a water-soluble form on P deficient soil, and that N use efficiency depended on soil having sufficient plant-available P. The delay in publishing these results is interesting. Lawes was busy between 1834 and 1842 developing his process for manufacturing water-soluble, single superphosphate, which he patented in 1842, and establishing a factory in London producing it commercially in 1843. But Lawes also must have realized that the results of his experiments in 1838–1839 supported the statement by Liebig in the first edition of his book that plants could not obtain sufficient N from the atmosphere to grow well (Liebig, 1840).

In the first edition of his book Liebig (1840) wrote: “Cultivated plants receive the same quantity of nitrogen from the atmosphere as trees, shrubs and other wild plants *but this is* ***not*** *sufficient for the purposes of agriculture”.* The crucially important phrase was changed in the third and fourth editions to: “…. *and this* ***is*** *quite sufficient for the purposes of agriculture”,* which was not supported by the evidence from Lawes’s early experiments*.* Lawes & Gilbert’s contribution to agricultural science owes much to Liebig’s change of “not” for “is”.

**Important early results from Broadbalk**

**Long-term experiments and environmental issues**

*Cadmium.* Concern about this carcinogenic element, which is added to soil in atmospheric deposition and in many phosphatic fertilizers, began in the late 1960s. Much added Cd is retained in soil from where it can transfer to the food chain. Johnston & Jones (1995) summarized some European data on Cd concentrations in crops and soils and related them to data from Rothamsted experiments. They also showed that the concentration of Cd in archived wheat and barley samples grown on Broadbalk and Hoosfield, respectively, on plots with and without increased Cd levels changed little over time, perhaps because translocation from stem to grain was limited. Nicholson *et al*. (1994) showed that the Cd concentrations in 5-year bulked hay samples from two plots on Park Grass, and the variations in Cd concentrations were shown to be due to addition of Cd from atmospheric deposition and P fertilizer, soil pH and herbage yield (Johnston & Jones, 1995). In general, the data showed that maintaining soil above pH_water_ 6 minimized the concentration of Cd in both the cereal grain and the herbage.

*Lead****.***  There was considerable interest in the effects of the reduction of lead (Pb) permissible in petrol from 0.4 to 0.15 g l^-1^ in January 1986 because the major source of Pb in the atmosphere in UK was vehicle emissions, and atmospheric deposition is the major source of Pb to agricultural soil in the UK (Jones *et al.*, 1991). Thus, Pb in and on vegetation is one pathway by which Pb can enter the food chain. Lead, in general, declined in samples of herbage from 1956 to 1988 from the Reference Plots at Rothamsted (Figure S1) with a sharp decline in the latter half of the 1980s. The variation between years strongly supports the need for observations and or measurements over a long period to be able to identify change accurately.


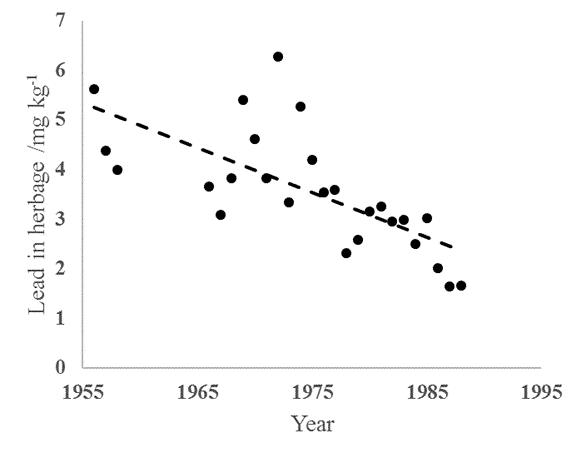


**Figure S1** Concentrations of lead in herbage, 1956–1988, for plots receiving NPK fertilizers. Reference Plots, Rothamsted. (From Jones *et al.*; 1991)

*Organic pollutants.*  Crop and soil samples from the archive have been analysed for organic pollutants including polynuclear aromatic hydrocarbons (PAHs), polychlorinated dibenzo–p–dioxins and –furans (PCDD/Fs), and polychlorinated biphenyls (PCBs), and the data were summarized by Jones *et al*. (1995 and references therein); all compounds that Lawes & Gilbert would not have heard about! One fascinating feature for the time trend data for ƩPCB concentrations is that the total burden for UK soils declined from *c*. 26 600 t in 1970 to *c*. 1500 t in 1990. Assuming that volatilization has been the only loss mechanism, outgassing from soil is potentially a major contributor to the atmospheric burden of PCBs and their long-range atmospheric transport (Harrad *et al*., 1994).

**Curve shifting**

Two further examples of how curves fitted to data collected over a limited number of years can be shifted to extend the period of change are:

*Microbial activity and* *SOM.*  Starting in 1942, FYM, FYM compost, sewage sludge and sludge and straw compost, with C:N ratios ranging from 9.5 to 13.8:1 were added at 37.5 and 75 t ha^-1^ fresh material each year to a sandy loam soil in the Woburn Market Garden experiment.. In 1960, the SOM with each treatment had a narrower C:N (10 to 11:1), and the linear relation between %OC and t ha^-1^ organic matter added accounted for 82% of the variance (Johnston *et al*., 2009, Figure 1). Sludge and sludge compost were not applied after 1960 and the FYM and FYM compost after 1967, and %OC declined in these soils. The eight decline curves were brought into coincidence by horizontal shifts (Figure S2) that were related only to the different starting contents of SOM and not to the different organic materials added. This suggests that the heavy metals in the sludge did not adversely affect the microbes decomposing the SOM although they did adversely affect the *Rhizobium* bacteria because clover yields were smaller on the sludge treated plot (Johnston *et al*., 1989).


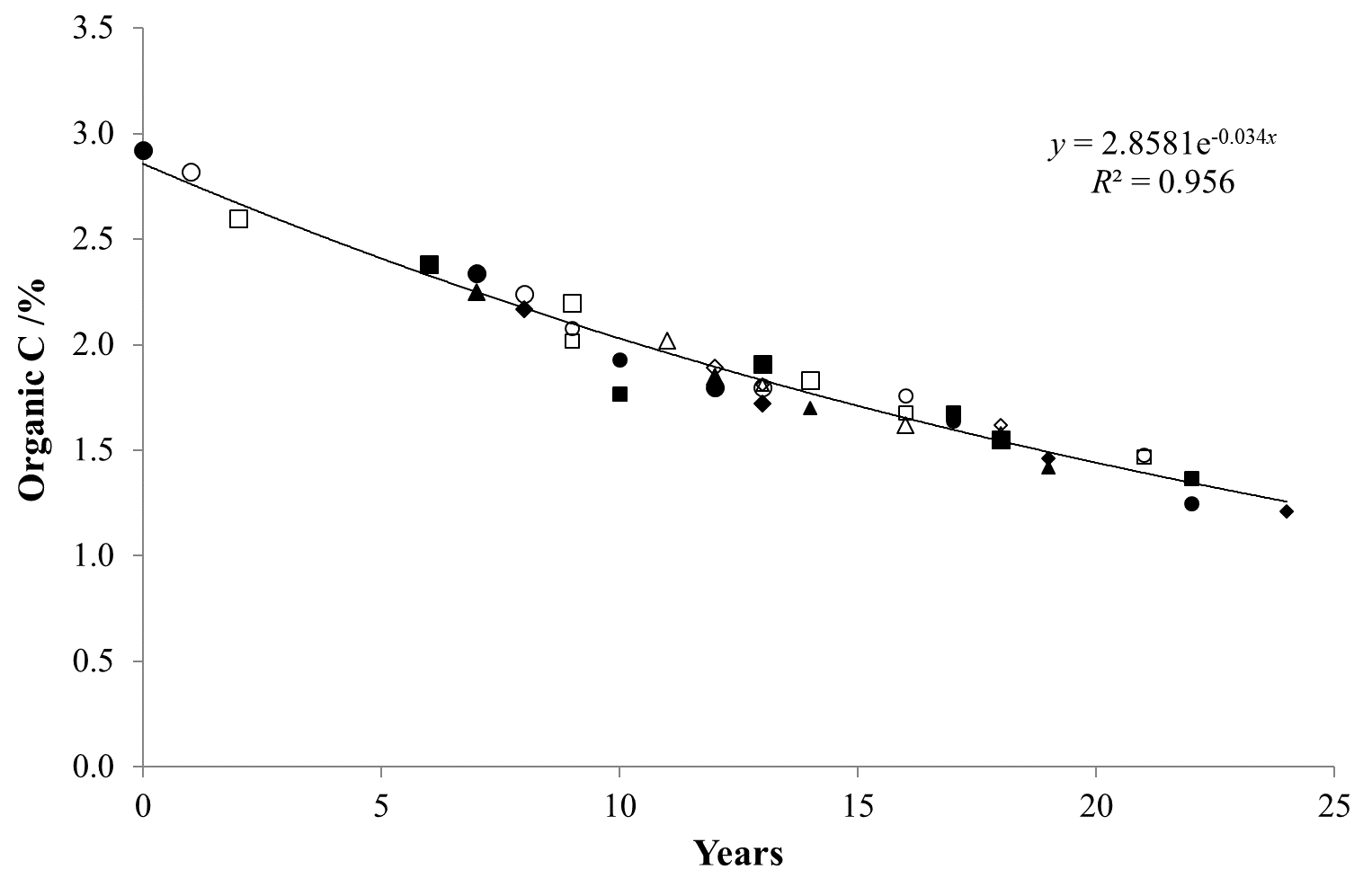


**Figure S2** Decline in organic C in the top 23 cm of soil. Symbols denote previous inputs of FYM, sewage sludge, FYM compost and sludge compost. After treatments stopped, soils were sampled over the next 5–12 years. The data could be shifted horizontally to fit a common exponential model. Market Garden experiment, Woburn. (Adapted from Johnston *et al.*; 1989).

*Decline in* *plant-available P in soil.*  Johnston *et al*. (2016) used data from long-term experiments to determine the length of time it will take for a P-enriched soil to decline to the critical level of plant-available P if P is no longer applied. For example, in 1967 there were eight soils with Olsen P ranging from 3 to 60 mg kg^-1^, no more P was applied, crops were grown, and Olsen P was measured in alternate years for 16 years. The eight P-decline curves (Figure S3a) were shifted horizontally to produce a unified decline curve (Figure S3b) from which it would take about nine years on this soil for Olsen P to decline by about half way to the lowest level.


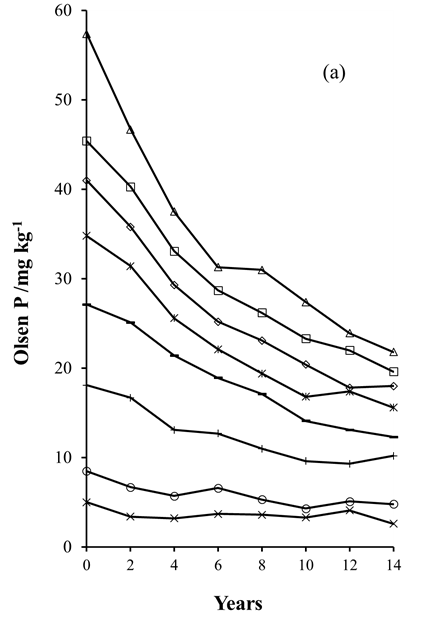


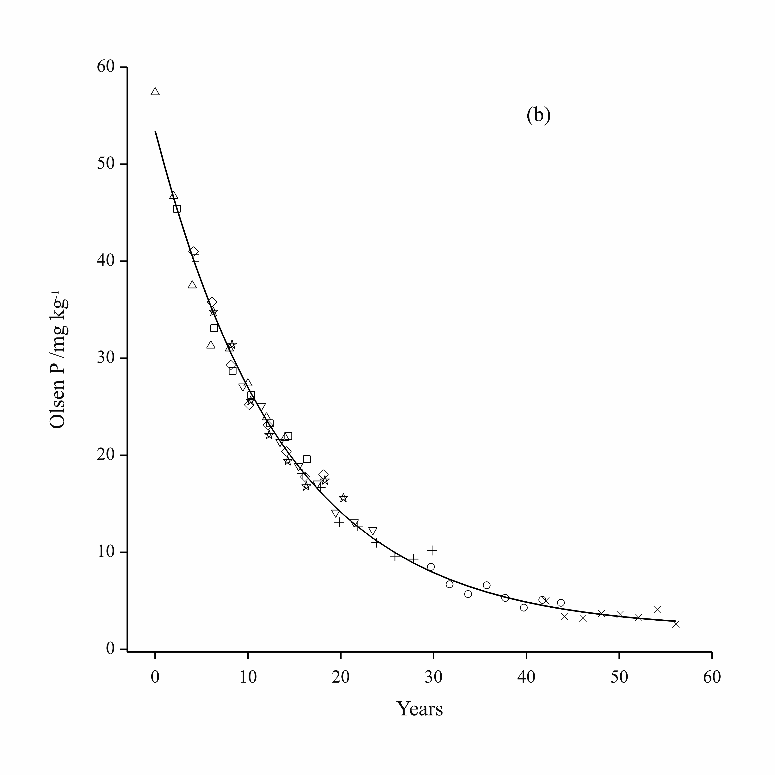


**Figure S3** Change in Olsen P on a sandy clay loam given no P for 14 years**:** (a) data for eight individual treatments and (b) an exponential decay curve fitted to the data for the eight treatments once the curves have been bought into coincidence by a series of horizontal shifts. Different symbols denote treatments which started at different concentrations of Olsen P. Rotation II experiment, Saxmundham. (From Johnston *et al.*, 2016).

**Ancillary experiments**

Analysis of herbage and soil from Park Grass for the stable isotope of sulphur, ^34^S, was used to assess the effect of sulphur dioxide (SO_2_) on sulphur cycling within the plant–soil system on Park Grass (Zhao *et al.*, 1998). Herbage S was positively correlated with total SO_2_ emissions in the UK whereas *δ*^34^S concentrations were negatively correlated with SO_2_ emissions reflecting the more negative *δ*^34^S values associated with anthropogenic S sources.

Two pathogens of wheat, *Phaeosphaeria nodorum* and *Mycosphaerella graminicola* have both been the cause of significant losses in yield at different times over the years. The DNA from these two pathogens was extracted from archived wheat straw samples from the Broadbalk experiment. The relative importance of one species compared to the other over time was strongly correlated with changes in SO_2_ emissions in the UK (Bearchell *et al*., 2005).

**Some of the challenges faced in maintaining the Woburn Ley–arable experiment.**

This comprehensive, complicated experiment was started in 1938 to test and compare crop yields in all-arable and ley–arable crop rotations. At that time, it was considered that alternating arable crops with periods of three to four years in leys, hence ley–arable cropping, could probably increase crop yields and soil fertility. The leys could be grasses and or clovers or forage legumes like lucerne (*Medicago sativa* L.). However, the many management changes needed over the 70-year period illustrate the need for careful monitoring of all aspects affecting soil fertility and crop yields, and responding appropriately to ensure the continuity of an experiment.

The initial and continuing component of this experiment is a comparison of four contrasted 5-year rotations that are repeated on the same plots, and with each phase of the rotation present each year. (The sequence of cropping is given in Johnston *et al*., 2017). In each rotation, three years of ‘treatment’ crops are followed by two years of ‘test’ crops the yields of which measured the effect of the treatment cropping. Table S3 shows the initial treatment and test crops in the four rotations.

In 1955, 18 years after the start of the experiment, the yields of potatoes grown as the sixth potato crop after grass ley and lucerne were 19.4 and 14.7 t ha^-1^, respectively, and as the eighth crop after arable (hay) and arable (roots) were 4.8 and 3.8 t ha^-1^, respectively. The cause of the large difference in yield was the build-up of potato cyst nematode (*Globodera rostochiensis*) where potatoes had been grown more frequently. To maintain the continuity and sustainability of the experiment sugar beet replaced potatoes as first test crop in 1956.

In 1951–52, the yields of potatoes following lucerne (25.2 t ha^-1^) were less than those following grazed ley (31.6 t ha^-1^) and only a little better than those in the all-arable rotation (21.8 t ha^-1^). Crop and soil analysis showed that the difference in yield after lucerne and ley was due to large amounts of potassium (K) removed in the lucerne and the depletion of soil K reserves. Initially, basal K applications were increased on plots growing lucerne, and later it was decided that it would be better to maintain an adequate level of plant-available K (121–180 mg kg^-1^ exchangeable K) in the top 25 cm (plough depth) soil on all plots to ensure an adequate supply of K within the topsoil.

After 1957, the yields of lucerne in the second and third years (5.77 and 5.26 t ha^-1^, respectively), were less than those of the second and third crops in 1944–1956 (7.09 and 7.91 t ha^-1^, respectively) even though more K was now being applied. The smaller yields were due to damage by stem eelworm (*Ditylenchus dipsaci*) and this problem was not resolved by fumigation of the soil and the use of fumigated seed, and lucerne was replaced by another legume, initially by sainfoin and later by clover.

After sugar beet replaced potatoes as first test crop it was grown for three 5-year rotations with a test of four rates of N. The best yields after the ley were little different to those in the arable rotations and this was due to the build-up of free-living nematodes *Longidorus* and *Trichodorus* in the plots that had been three years in grazed ley. Thus, in 1971, potatoes, with and without soil fumigation to control potato cyst nematode, replaced sugar beet as first test crop and in all four rotations fumigation consistently increased yields from 6.6 to 10.5 t ha^-1^.

To simplify the management of the experiment, the leys were changed in 1973. Initially, they were a grass–clover ley with a small amount of N and grazed by sheep and lucerne. Following problems with nematodes affecting lucerne and grazing small plots with sheep, the leys were changed to an all-grass ley with fertilizer N and a grass–clover ley without fertilizer N and 10–15% clover in the seeds mixture. With increasing farmer interest in cereal crops rather than root crops, winter wheat became first test crop in 1976 followed by spring barley as second test crop.

**Experiments that were stopped**

*Woburn permanent wheat and barley experiments.* Woburn Experimental Station was started by the Royal Agricultural Society of England in 1876 with the principal aim of checking the tables of compensation payable to a tenant leaving a farm for the unexhausted manurial value of purchased feed-stuffs (Johnston, 1977). Lawes and Gilbert served in an advisory capacity and at their suggestion experiments on winter wheat and spring barley grown continuously, like those at Rothamsted, but with simplified treatments, were started in 1876 with winter wheat and in 1877 with spring barley to see which manurial treatments would best maintain yield. The soil, a sandy loam with a pH *c*. 6 in 1876, acidified rapidly with ammonium sulphate and barley yields declined dramatically. Although applying chalk had a beneficial effect, a comprehensive test of fresh chalk and its residual effect could not be included on the existing plots. Thus, as neither the original aims nor the factors that should be tested could be investigated effectively the experiments were stopped in 1966. After further correction of soil pH, the increase in yields from the residues of P and K was measured for a few years on the top third of the site whereas what was intended to be a long-term P experiment was started on the bottom third. Soon after this experiment had started, very intense rainfall resulted in surface runoff, severe gully erosion and the transfer of soil between plots and the experiment had to be abandoned.

*The rotation experiments.* In the 1930s arable crops were grown in rotation as a way of controlling weeds, pest and diseases, and rotation experiments started in this period used the concepts of multifactorial experiments and randomization and replication developed by Fisher. Four arable crop experiments, testing 2-, 3-, 4- and 6-course rotations were started with a mix of root crops and cereals, to provide information on best fertilizer practises. With more combinable crops being grown in the 1960s, the value of the data declined and the experiments were gradually stopped. However, there was an unexpected value in the 4-course rotation in which superphosphate and untreated rock phosphate had been compared in the year of application and the four residual years. Superphosphate always gave larger yields than rock phosphate but this difference could not be explained when the soils were analysed with acid extractants for plant-available P. But Olsen’s reagent when tested on soil samples in the late 1950s not only separated plots with superphosphate and rock phosphate in terms of the availability of the P in the year of application but in the four residual years also, and this led to the use of this method at Rothamsted (Warren & Johnston, 1965).

**Photographs**


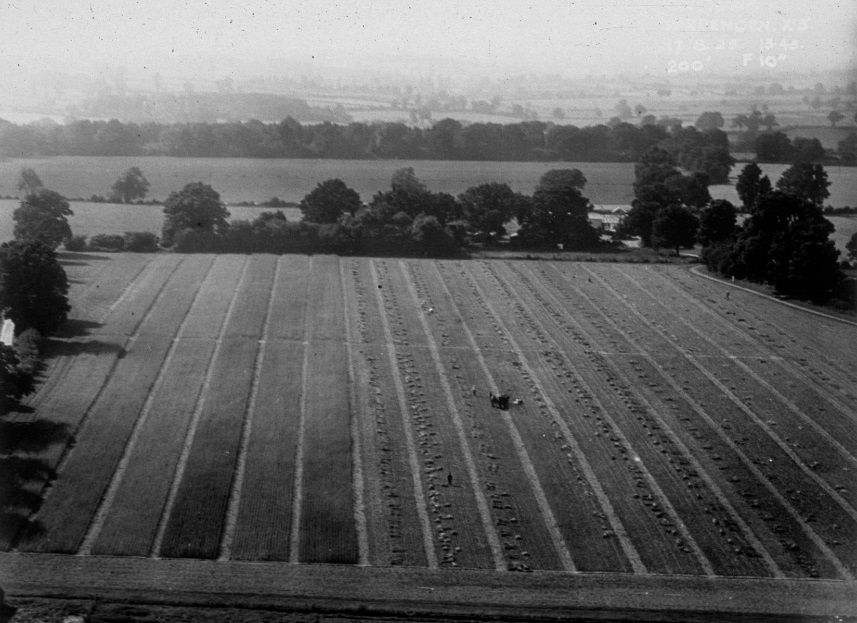


**Photograph 1.** Aerial view of the Broadbalk Wheat experiment in August 1925, part-way through harvest. Most of the treatment strips are *c.* 300 m long and 6 m wide. The following year the experiment was divided into five sections; these were then bare fallowed in turn to control weeds.


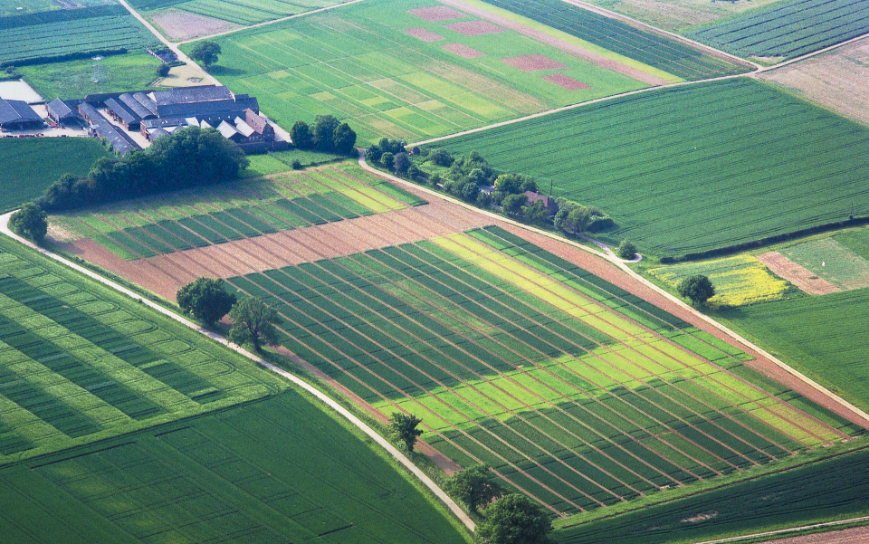


**Photograph 2.** Aerial view of the Broadbalk Wheat experiment in early summer 2003. In 1968, the experiment had been divided into the 10 sections seen in the photograph (section 0 at the top and section 9 at the bottom of the field). Five of the sections are in continuous wheat; on the others wheat is grown in rotation. From 1996–2017 the 5-course rotation was winter oats (*Avena sativa* L.), forage maize (*Zea mays* L.), wheat, wheat, wheat. The oats are grown without fertilizer N or FYM (section 7) whilst the maize has only recently been drilled (section 2).


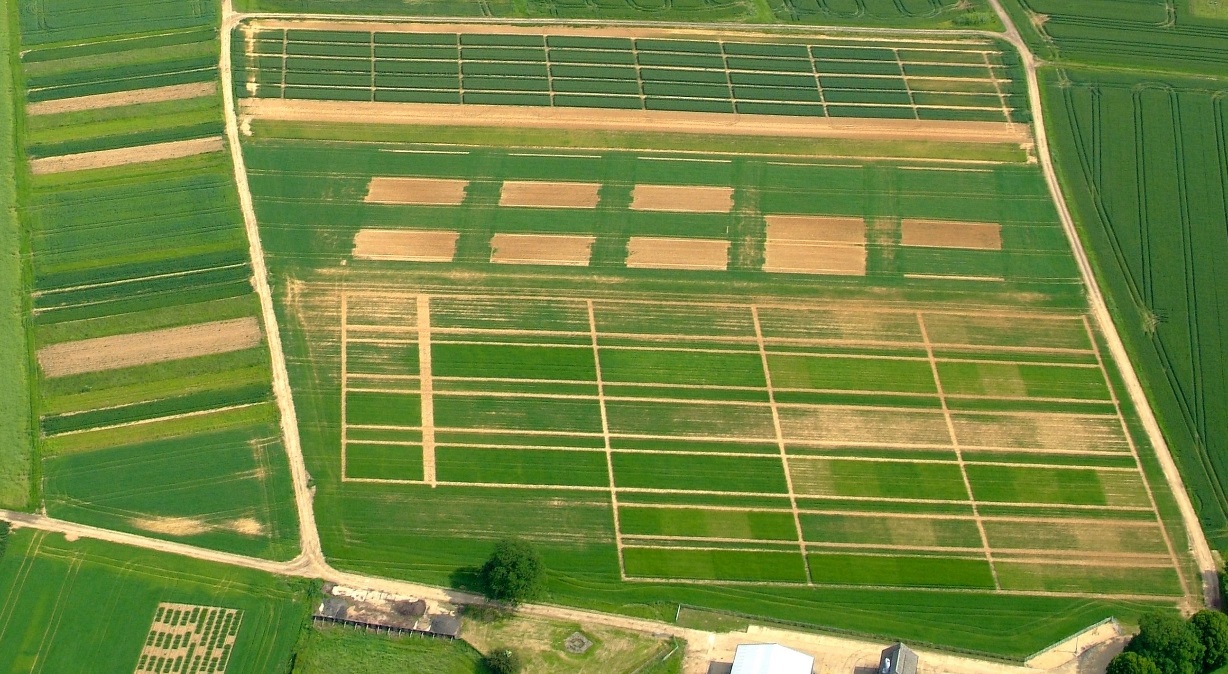


**Photograph 3.** Aerial view of the Hoosfield Barley experiment started in 1852 (foreground), the Exhaustion Land experiment started in 1856 (top) and the Fosters Ley–arable experiment which started in 1949 (left). Taken early summer 2012.


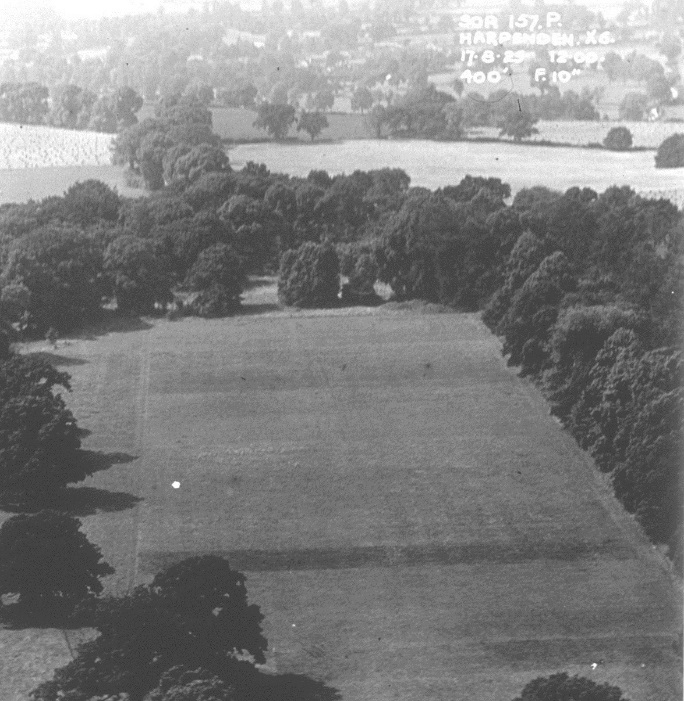


**Photograph 4.** Aerial view of the Park Grass experiment in August 1925 when most plots had been divided to test the application of chalk. Half of each plot (on the right-hand side of the photograph) received no chalk and half received 4 t CaCO_3_ ha^-1^ every four years.


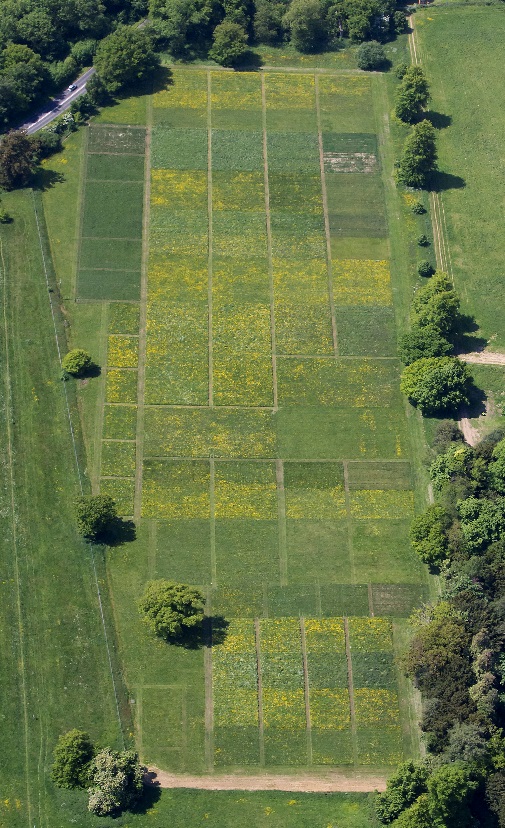


**Photograph 5.** Aerial view of Park Grass in 2001. In 1965, most plots had been divided into four subplots. Looking from right to left on the photograph, the d subplots did not receive chalk whilst the c, b and a subplots received different amounts of chalk to maintain the soil (0–23 cm) at pH 5, 6 and 7. The plots on the far left-hand side were used to test a different liming scheme from 1920–1964.


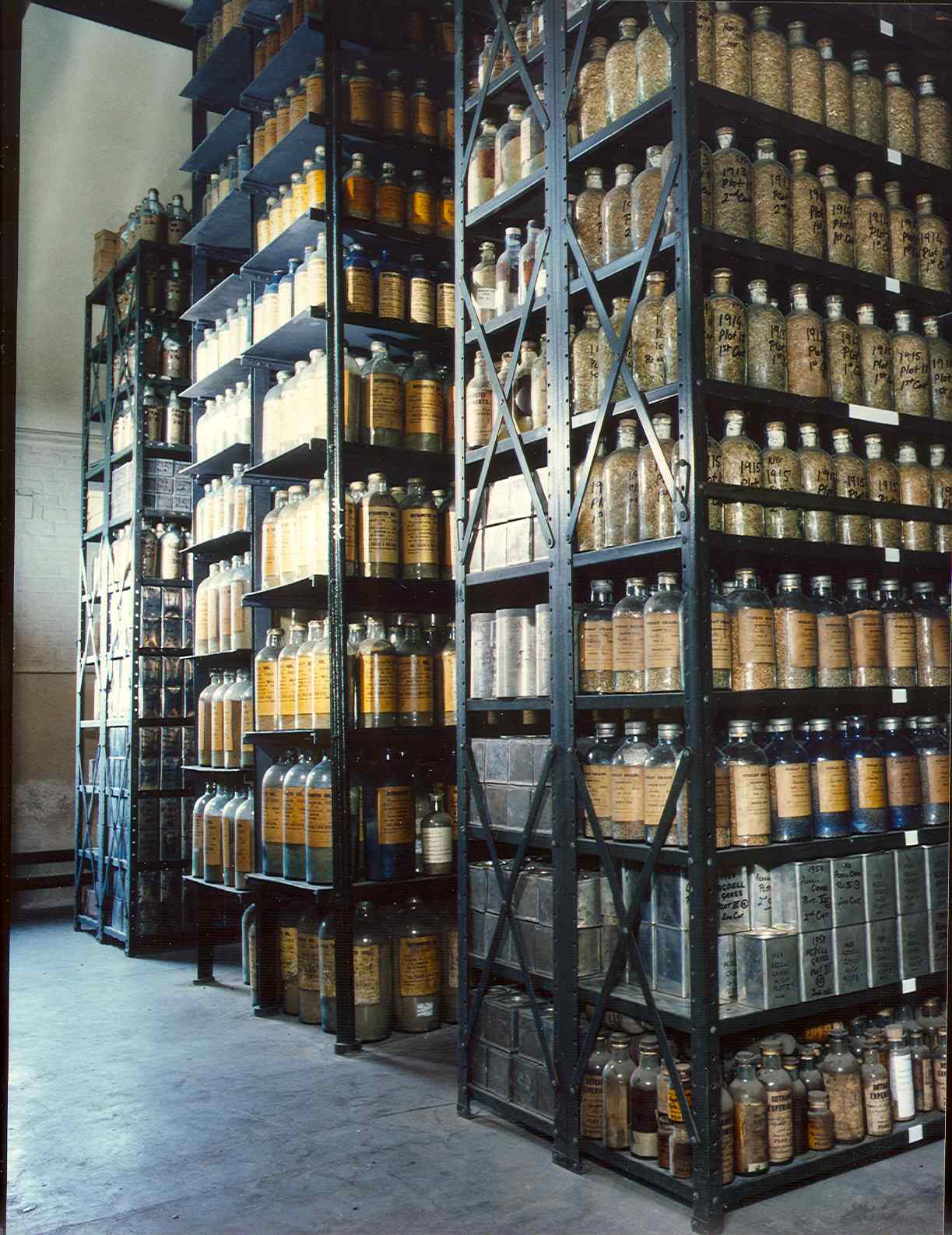


**Photo 6.** The old Rothamsted Sample Archive. From 1948 to 2008 the Sample Archive was housed in out-buildings at Rothamsted Manor. But, by 2008, there was no room for further samples.


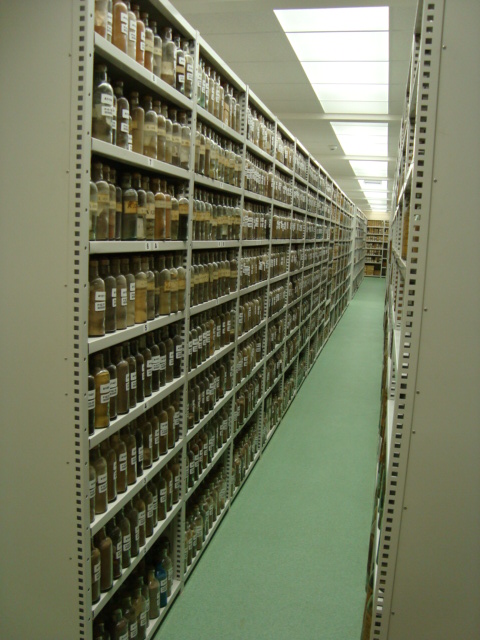


**Photo 7.** The new Rothamsted Sample Archive. In 2008, the Sample Archive was moved to a temperature controlled facility with space for samples for *c.* 30 years. At present, the Archive contains *c.* 300 000 samples.

**References**

Bearchell, S.J., Fraaije, B.A., Shaw, M.W. & Fitt, B.D. 2005. Wheat archive links long-term fungal pathogen population dynamics to air pollution. *Proceedings of the National Academy of Sciences*, **102**, 5438–5442.

Harrad, S.J., Sewart, A.S., Alcock, R.E., Boumphrey, R., Burnett, V., Duart-Davidson, R. *et al*. 1994. Polychlorinated biphenyls (PCBs) in the British environment: sinks, sources and temporal trends. *Environmental Pollution,* **85,** 131–147.

Johnston, A.E. 1977. *Woburn Experimental Farm: A Hundred Years of Agricultural Research*. Lawes Agricultural Trust, Harpenden UK.

Johnston, A.E. & Jones, K.C. 1995. *The Origin and Fate of Cadmium in Soil*. Proceedings **366***.* The International Fertiliser Society, Peterborough.

Johnston, A.E., Poulton, P.R. & Coleman, K. 2009. Soil organic matter: Its importance in sustainable agriculture and carbon dioxide fluxes. *Advances in Agronomy*, **101**, 1–57.

Johnston, A.E., Poulton, P.R., White, R.P. & Macdonald, A. J. 2016. Determining the longer-term decline in plant-available soil phosphorus from short-term measured values. *Soil Use and Management* **32,** 151–161.

Johnston, A.E., McGrath, S.P., Poulton, P.R. & Lane, P.W. (1989). Accumulation and loss of nitrogen from manure, sludge and compost: long-term experiments at Rothamsted and Woburn. In: *Nitrogen in Organic Wastes Applied to Soils* (eds J.A.A. Hansen & K. Henriksen), pp. 126–139. Academic Press, London.

Johnston, A.E., Poulton, P.R., Coleman, K., Macdonald, A.J. & White, R.P. 2017. Changes in soil organic matter over 70 years in continuous arable and ley–arable rotations on a sandy loam soil in England. *European Journal of Soil Science,* **68**, 305–316.

Jones, K.C., Symon, C.J., Taylor, J.P.L., Walsh, J. & Johnston, A.E. 1991. Evidence for a decline in rural herbage lead levels in the UK. *Atmospheric Environment,* **25A**, 361–369.

Jones, K.C., Johnston, A.E. & McGrath, S.P. 1995. Historical monitoring of organic contaminants in soils. In: *Long-term Experiments in Agricultural and Ecological Sciences* (eds. R.A. Leigh & A.E. Johnston), pp. 147–163. CAB International, Wallingford.

Lawes, J.B. 1842. Ammoniacal manure. *The Gardeners’ Chronicle.* London. p. 221.

Lawes, J.B. 1843. Ammonia. *The Gardeners’ Chronicle.* London. p. 692.

Liebig, J. von. 1840, 1843. *Organic Chemistry in its Application to Agriculture and Physiology,* 1st and 3rd/4th Edition. Taylor and Walton, London.

Nicholson, F.A., Jones, K.C. & Johnston, A.E. 1994. Effect of phosphate fertilizers and atmospheric deposition on long-term changes in the cadmium content of soils and crops. *Environmental Science & Technology,* **28,** 2170–2175.

Warren, R.G. & Johnston, A.E. 1965. Notes on the use of soil analysis for estimating available P in Rothamsted soils. In: *Soil Phosphorus.* Technical Bulletin 13. Ministry of Agriculture, Fisheries and Food. pp. 30-37. HMSO. London.

Zhao, F.J., Spiro, B., Poulton, P.R. & McGrath, S.P. 1998. Use of sulfur isotope ratios to determine anthropogenic sulfur signals in a grassland ecosystem. *Environmental Science & Technology,* **32**, 2288-2291.
